# Evaluation of Single and Joint Toxicity of Perfluorinated Carboxylic Acids and Copper to Metal-Resistant *Arthrobacter* Strains

**DOI:** 10.3390/ijerph16010135

**Published:** 2019-01-07

**Authors:** Yanping Cai, Haiyan Chen, Huilun Chen, Haiqing Li, Shuo Yang, Fei Wang

**Affiliations:** 1 School of Energy and Environmental Engineering, and Beijing Key Laboratory of Resource-Oriented Treatment of Industrial Pollutants, University of Science and Technology Beijing, 30 Xueyuan Road, Haidian District, Beijing 100083, China; cyp38948@foxmail.com (Y.C.); 13225736627@163.com (H.L.); tdchys@163.com (S.Y.); wangfei6699@aliyun.com (F.W.); 2 State Key Laboratory of Environmental Criteria and Risk Assessment, Chinese Research Academy of Environmental Science, Beijing 100012, China; chenhy@craes.org.cn

**Keywords:** perfluorinated carboxylic acids (PFCAs), perfluorooctanoic acid (PFOA), microcalorimetry, carbon chain, joint toxicity

## Abstract

Perfluorocarboxylic acid compounds (PFCAs) and copper have been regarded as ubiquitous environmental contaminants in aquatic ecosystems worldwide. However, data on their possible joint toxic effects on microorganisms are still lacking. To study the combined effects of four PFCAs with different carbon chain lengths and copper, a series of experiments were conducted to explore the acute toxicity of these PFCAs in the absence and presence of copper on a metal-resistant *Arthrobacter* strain GQ-9 by microcalorimetry. The thermokinetic parameters, including growth rate constant (*k*), inhibitory ratio (*I*), and half inhibitory concentration (*IC*_50_), were calculated and compared using the data obtained from the power-time curves. Our work revealed that GQ-9 is more resistant to perfluorooctanoic acid (PFOA) than *Escherichia coli*. The single and joint toxicity of PFCAs with copper are dose- and carbon chain length-dependent. The longer the carbon chain length of PFCAs, the higher the toxicity. In addition, PFCAs interacted synergistically with copper. This work could provide useful information for the risk assessment of co-exposure to perfluorinated compounds and heavy metals in natural environments.

## 1. Introduction

Perfluorinated compounds (PFCs) are a class of synthesis chemicals with unique physical and chemical characteristics that have made them extensively used in major industries and consumer products for over 60 years [1]. The widespread use and superior properties of PFCs have contributed to their ubiquitous occurrence in the global environment [2]. The perfluorocarboxylic acids (PFCAs) are a type of PFC whose structures are organized by carboxylate at one end of their molecules and have all of the hydrogens substituted with fluorine. This kind of structure imparts PFCAs with water and oil repellency, as well as thermal stability, all of which results in them being persistent pollutants [3]. Among PFCAs, perfluorooctanoic acid (PFOA) has been identified as the predominant pollutant in the blood sera of employees in the fluorochemical manufacturing industry; its concentration in the blood of employees can reach up to 114 μg/mL [4,5]. PFCAs have been proven to have bioaccumulation and biomagnification capacity via food chains and can cause peroxisome proliferation and affect mitochondrial, microsomal, and cytosolic enzymes and proteins involved in lipid metabolism [2,6,7]. Their global occurrence, persistence in the environment, and bioaccumulation in biota have aroused international concerns about the possible toxic effects of PFCAs.

Contamination with heavy metals in freshwater ecosystem has always been of great concern, since they are bioaccumulative, non-biodegradable, and toxic to aquatic biota. Copper (Cu), as one of the essential nutrients, is maintained in homeostasis in the human body [8]. However, exceeding the recommended copper intake can cause toxic effects such as hemolysis, jaundice, and even death [9]. Copper has been extensively detected in aquatic environments that have been impacted by runoff from mining, waste emissions, and application of pesticides, with concentrations recorded at 152 μg/L [10].

Currently, the toxicological studies on PFCAs mostly focus on plants, aquatic organisms (phytoplankton, zooplankton, fish, etc.), mammals such as mice, tissues, organs (liver, kidney, etc.), and cells (endothelial cells, liver cells, fat cells, etc.) [11,12,13,14,15,16,17]. However, toxicological studies regarding microorganisms are very limited [18].

Furthermore, PFCAs always coexist with various contaminants in real aquatic environments. Zheng, et al. [19] investigated some sediment samples collected from the Dayan River and found that heavy metals co-occur with PFCAs and they share a common anthropogenic source. Although most of these compounds are present at low concentrations, many of them raise considerable toxicological concerns, particularly when present as components of complex mixtures, which may interact and pose associated effects on aquatic environments [20]. However, little research has been conducted to show the combined toxic effects of PFCAs and heavy metals [21,22,23,24]. Rodea-Palomares, Leganés, Rosal and Fernández-Piñas [23] found that the interaction of PFOA with Cd was mostly antagonistic to a bioluminescent cyanobacteria. Qu, Liu, Wang and Wang [24] reported that perfluorooctane sulfonate (PFOS) and Cd mixtures mainly showed simple addition and antagonism to the freshwater oligochaete at the test concentrations. Unfortunately, previous studies have failed to evaluate the joint effects of PFCs and copper on microorganisms thoroughly, leaving the corresponding joint toxicity in some special microorganisms, such as thermophilic bacteria, alkali-resistant bacteria, and metal-tolerant bacteria, which would stimulate the research on the microbial degradation of PFCAs, to be unknown.

An *Arthrobacter* strain GQ-9, which was isolated from a heavy metal polluted soil and had a resistant capability to Cu^2+^ with a maximal tolerable concentration (MTC) of 110 mg/L [25], was chosen as the test organism. Microcalorimetry measures the heat flow of biological process, which is proportional to the rate at which a given chemical or physical process takes place. The microcalorimetric technique is a simple, straight-forward method that can be employed to obtain a variety of kinetic and thermodynamic information on metabolism of microorganisms [18,26]. In the present study, a systematic experimental approach was used to evaluate the single and joint toxicity of PFCAs and Cu to a metal-resistant bacteria by considering the effect of carbon chain length (4, 6, 8, 10). The current work could provide updated knowledge about the combined toxic effects of PFCAs and Cu to microorganisms, which can facilitate our understanding on the potential risks of coexisting pollutants in natural aquatic environments.

## 2. Materials and Methods

### 2.1. Microorganisms and Materials

The *Arthrobacter* strain GQ-9, obtained from our previous study [25], was used as the test organism for this study. The Luria-Bertani (LB) medium that incubated the *Arthrobacter* strain was prepared by dissolving 10 g tryptone, 5 g yeast extract, and 10.0 g NaCl in 1.0 L of deionized water at pH 7.2. It was then sterilized in a high-pressure steam at 121 °C for 30 min.

The perfluorobutyric acid (PFBA), perfluorohexanoic acid (PFHxA), PFOA, and perfluorodecanoic acid (PFDA) were purchased from Sigma-Aldrich Corporation (St. Louis, MO, USA). The buffer solution was prepared with sodium dihydrogen phosphate (NaH_2_PO_4_·2H_2_O) and sodium monohydrogen phosphate (Na_2_HPO_4_·2H_2_O) (FuChen Chemical Reagent Factory in Tianjin, China) to maintain a neutral pH of the PFCA solutions. The copper sulfate (CuSO_4_·5H_2_O) was obtained from FuChen Chemical Reagent Factory in Tianjin, China.

### 2.2. Experimental Procedure

Stock culture (−70 °C) was reactivated at 28 °C with the LB medium in an orbital shaker (150 rpm) for 12 h to get enough inoculums. Then, the cultures inoculated for microcalorimetric measurement were transferred into 4.0-mL stainless steel ampoules and were loaded with 2.0 mL LB medium. For all experiments, cell suspension was used at a concentration of 2 × 10^8^ cells/mL and samples were incubated at 28 °C unless otherwise stated.

### 2.3. GQ-9 Growth Curve

The bacterial growth curve under combined exposure of PFOA and copper was carried out in LB medium for determination of the microbial growth in an ultraviolet-visible (UV-VIS) spectrophotometer (Model 752; Lengguang Technology, Shanghai, China) at λ_600_ nm. All experiments were conducted in triplicate.

### 2.4. Single and Joint Toxicity Test

The tests were performed using a TAM III multi-channel thermal activity microcalorimeter from TA Instruments, New Castle, DE, USA. In this study, the microcalorimetric measurements were based on the isothermal ampoule method. The power-time curve was obtained by using 4.0mL stainless steel ampoules that were hermetically closed by Teflon sealing discs to prevent evaporation and energy loss. The toxicity tests were performed in ampoules containing 2.0 mL of culture medium incubated with microbes and various doses of the PFCA solutions in the absence and presence of 5 mg/L Cu^2+^. A computer continuously recorded the power-time curves of the growth of GQ-9.

The microbial growth rate constant (*k*, min^−1^) was obtained from power-time curves and calculated by Equation (1):

(1) $$\ln P_{t}=\ln P_{0}+k\left(t-t_{0}\right)$$

where $P_{t}$ (μW) is the heat output power at time *t* (min) and $P_{0}$ (μW) is the heat output power at time $t_{0}$ (min).

The inhibitory ratio *I* was obtained using Equation (2):

(2) $$I=\frac{k_{0}-k_{C}}{k_{0}}\times 100\%$$

where $k_{0}$ is the rate constant of the blank and $k_{C}$ is the rate constant of GQ-9 activity inhibited by chemicals with concentration *C*. When *I* is 50%, the corresponding concentration of the chemicals is indicated as *IC*_50_ [26].

### 2.5. Statistical Analyses

The growth rate constant (*k*) was obtained through three parallel experiments. The analysis of variance (ANOVA) method was used for statistical analysis, with *p* ≤ 0.05 considered to be a significant level of difference between means with correction factors. Data are presented as arithmetic means ± standard deviations (SDs).

## 3. Results and Discussion

### 3.1. The Optical Density of GQ-9 Growth

**Figure 1:**
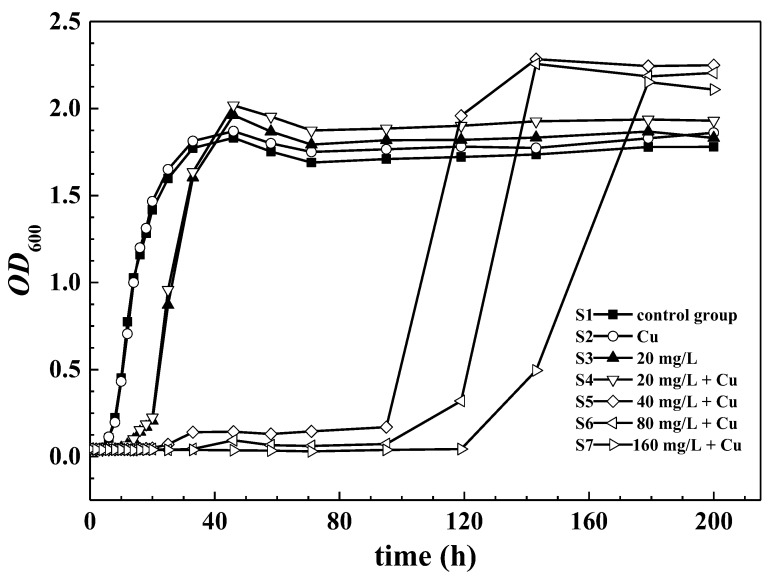
The optical density of GQ-9 when exposed to 5 mg/L Cu^2+^ and different concentrations of perfluorooctanoic acid (PFOA).

The growth of *Arthrobacter* strain GQ-9 in LB medium under different concentrations of PFOA was studied. As shown in Figure 1, S1 (control group) and S2 (individual Cu) indicate that the 5 mg/L copper had a slight positive effect on the metabolism of GQ-9, as it has a resistant capability to Cu [25]. With the addition of PFOA, the adaptation phase of *OD*_600_-time curve increases from 10 to 120 h with the concentration ranging from 20 to 160 mg/L. Furthermore, after the adaptation phase, S5, S6, and S7 begin to rise and their maximum values are all higher than those of the lower PFOA dose curves. As of the writing of this paper, no studies have investigated whether PFOA at high concentrations (>40 mg/L) would boost the growth of bacteria. In light of that, PFOA may bind to the copper ion at their carboxylic groups in the adaptation phase, altering the bioavailability, thereby decreasing the toxicity of PFOA [27]. However, the mechanism of GQ-9 resistant to PFOA requires further exploration.

### 3.2. Individual Toxicity of PFCAs to GQ-9 by Microcalorimetry

**Figure 2:**
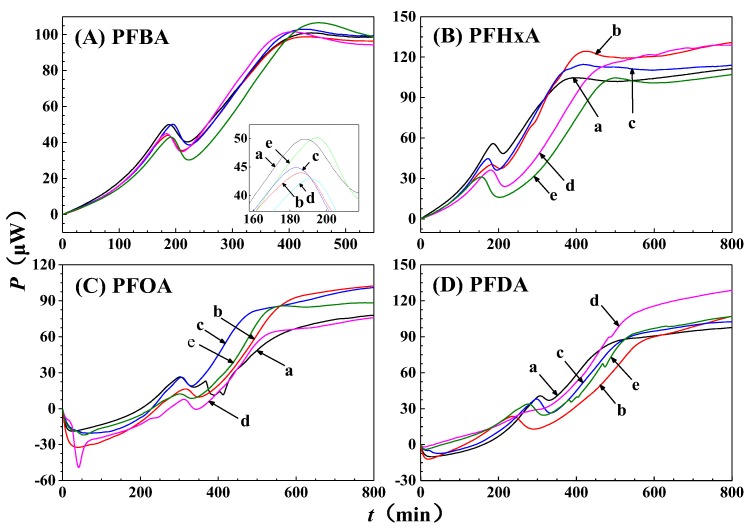
The power-time curves of GQ-9 grown at different concentrations of perfluorocarboxylic acid compounds (PFCAs) in Luria-Bertani (LB) medium. (**a**) 0 mg/L; (**b**) 20 mg/L; (**c**) 40 mg/L; (**d**) 80 mg/L; and (**e**) 160 mg/L.

**Figure 3:**
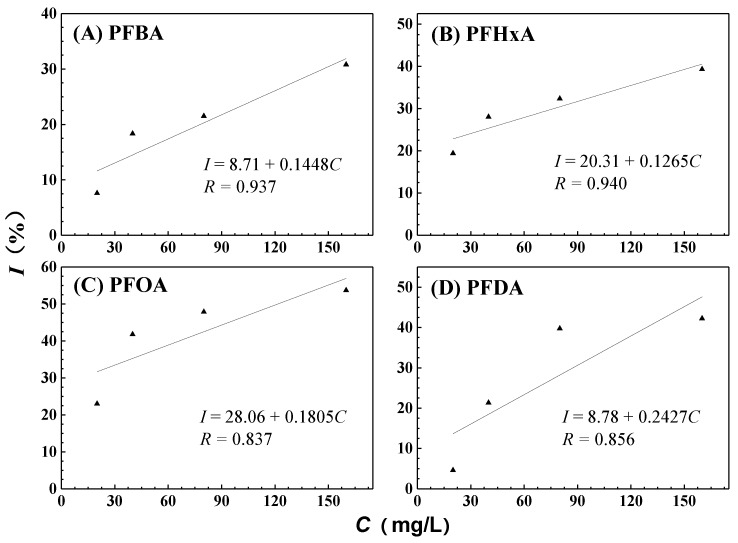
Dose-response relations for single toxicities of PFCAs. PFBA: perfluorobutyric acid; PFHxA: perfluorohexanoic acid; PFDA: perfluorodecanoic acid.

The power-time curves for the growth of GQ-9 in the presence of different concentrations of PFCAs are shown in Figure 2. The growth rate constant (*k*) of the microbial growth reaction can be calculated from the power-time curves according to Equation (1). Inhibitory ratio (*I*) was calculated on the basis of *k* using Equation (2). The *IC*_50_ value of PFCAs were determined using regression equations in Figure 3. The *k*, *I*, and *IC*_50_ of GQ-9 grown in the presence of various PFCAs are summarized in Table 1. Among these PFCAs, PFOA is the most toxic species, with an *IC*_50_ of 122 mg/L against GQ-9. PFDA and PFHxA exhibit moderate toxicity, with *IC*_50_ values of 170 mg/L and 235 mg/L, respectively. PFBA has the lowest toxicity, with an *IC*_50_ of 285 mg/L. It is interesting to note that all concentrations of PFCAs can inhibit the growth of GQ-9, as shown in Table 1. In a previous study, Liu, Zhang, Yang, Zhu and Lin [22] proposed that the *IC*_50_ of PFOA to *Escherichia coli* was 10.6 mg/L, while Chen, Yao, Wang, Cai and Liu [18] explored the toxic effect of PFOA against *Pseudomonas putida* and determined an *IC*_50_ of 100 mg/L; both of these *IC*_50_ value are much smaller than that of PFOA to GQ-9. The comparison shows that GQ-9 is more resistant to PFOA than *Escherichia coli* and *Pseudomonas putida*; this suggests that it could be an important model organism for evaluating eco-toxicity and investigating toxic mechanisms at the cellular level.

In this study, the toxicity order could be tentatively proposed as PFOA > PFDA > PFHxA > PFBA, according to the order of *IC*_50_ values. Actually, our present results were in accordance with the previous findings of Buhrke, et al. [28], who examined the cytotoxic effects of the different PFCA homologs on human liver cells through *IC*_50_ values and relative viability and came to the conclusion that PFCA compounds with a short carbon chain length, such as PFBA or PFHxA, seem to be less toxic than those with longer carbon chain lengths. The finding of PFOA being more toxic than PFBA was also described in the study by Hagenaars, Vergauwen, De Coen and Knapen [11]. In addition, Nobels, et al. [29] evaluated PFCAs with a bacterial multiple endpoint reporter assay for responses in oxidative damage and membrane damage, and their results demonstrated that inductions of stress responsive genes occur in the order of PFOA > PFDA > PFHxA > PFBA in terms of oxidative damage, and PFOA > PFBA, PFHxA, PFDA in terms of membrane damage.

Furthermore, a study on PFCAs by Kleszczyński, et al. [30] revealed a chain length-half maximal effective concentration (*EC*_50_) dependence on human colon carcinoma (HCT116) and estimated that values of *EC*_50_ decreased with elongation of C-F chain (PFHxA > PFOA > PFDA). Hoke, et al. [31] conducted short-term aquatic toxicity tests to evaluate the acute toxicity of PFCs and found that aquatic toxicity generally decreased as the number of fluorinated carbons decreased from 12 to 8. The same toxicity ranking of PFDA and PFOA was also observed by Liu, et al. [32] and Buhrke, Kibellus and Lampen [28]. Blaine, Rich, Sedlacko, Hyland, Stushnoff, Dickenson and Higgins [7] showed that there is differential uptake into plants based on chain length and that PFCAs with shorter chains are taken up more readily. PFOA and PFDA are known as peroxisome proliferators [33], and PFDA has more extensive toxicity compared to PFOA due to its higher accumulation in the target spot, which suggested that the accumulation of compounds in aqueous environments cannot account for the toxicity ranking of PFDA and PFOA. Thus, the specific toxic mechanism differences between two compounds need more in-depth research.

### 3.3. Joint Toxicity of PFCAs and Copper to GQ-9 by Microcalorimetry

**Figure 4:**
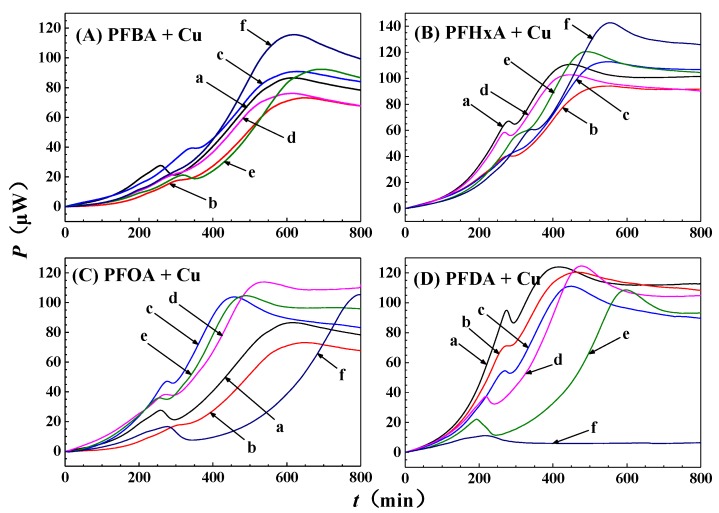
The power-time curves of GQ-9 growth at different concentrations of PFCAs with the addition of 5 mg/L Cu^2+^ in LB medium. (**a**) 0 mg/L; (**b**) 20 mg/L; (**c**) 40 mg/L; (**d**) 80 mg/L; and (**e**) 160 mg/L.

**Figure 5:**
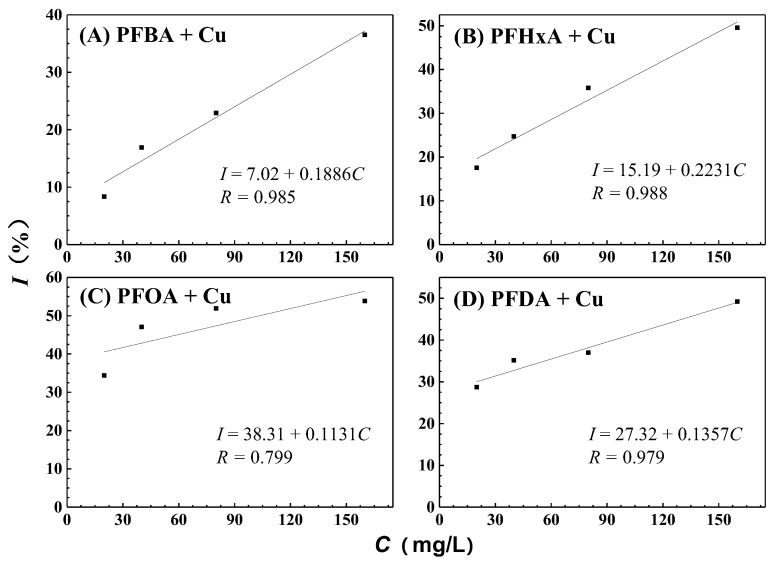
Dose-response relations for joint toxicities of PFCAs and copper.

The power-time curves for the growth of GQ-9 in the presence of different concentrations of PFCAs and copper are shown in Figure 4. Figure 5 shows that there is a markedly positive linear relationship between the inhibitory rates and the concentrations of PFCAs. The corresponding regression equations in Figure 5 were used to determine the *IC*_50_ values of different mixtures and are presented in Table 2. When GQ-9 was exposed to 5 mg/L Cu^2+^ alone, the inhibitory ratios were 7.41 (±6.95)%, thus indicating a slightly inhibitory impact on bacteria growth. With the addition of copper, PFOA exhibits the most toxicity, with an *IC*_50_ of 103 mg/L against GQ-9. PFDA and PFHxA are moderately toxic with *IC*_50_ values of 167 and 156 mg/L respectively, and PFBA has the lowest toxicity with an *IC*_50_ of 228 mg/L. The joint toxicity order is similar to the single toxicity order, which means that the toxicity of PFCAs are carbon chain length-dependent [28,29].

Moreover, the *IC*_50_ values of the combined exposure experiments are all lower than the single exposure experiments (Table 1 and Table 2), which indicates that the joint toxicity of PFCAs and copper is higher than the single toxicity of PFCAs, thus providing evidence of a synergistic effect between copper and PFCAs. The joint toxicity of PFOS/PFOA and copper to *Carassius auratus* was analyzed using oxidative stress biomarkers, with the results suggesting the existence of synergistic effects [21]. The most plausible explanation for the interaction of PFCAs with heavy metals could be the stabilization of the metals through either complexation [34] or counter-ion exchange with the negatively charged surfactants at the assay pH [35]. A rather small p*K*_a_ value would make more than 99% of the compound occur in its anionic form under most environmental conditions, and with the increase of CH_2_ units in the carbon chain, the p*K*_a_ of PFCAs decrease [36], thus the aqueous copper would more easily be stabilized by long carbon chain compounds. However, the differences in toxic interaction could be related to the test species used.

## 4. Conclusions

The current study was conducted to evaluate the single and joint toxicity of PFCAs and Cu towards a heavy-metal-resistant bacterium GQ-9. The toxicity of PFCAs can be simply represented by *IC*_50_. Our work reveals that GQ-9 is more resistant to PFOA than *E. coli*. Furthermore, the single and joint toxicities of the investigated PFCAs are dose-dependent and carbon chain length-dependent, in the order of PFOA > PFDA or PFHxA > PFBA. The PFCAs with longer carbon chains are more toxic than shorter ones, and the possible joint effects are proposed to be due to synergistic effects. However, in light of the chemical complexities of natural aquatic environments, further studies are needed to investigate the joint-action toxicity and underlying mechanisms of PFCAs and other pollutants on multiple aquatic organisms after chronic exposure.

## Figures and Tables

**Table 1:** Effect of single exposure to PFCAs in *k*, *I*, and *IC*_50_.

Sample	PFCAs (mg/L)	*k* (×10^−2^ min^−1^)	*I* (%)	*IC*_50_ (mg/L)
PFBA	0	4.74 ± 0.05	0	285
	20	4.38 ± 0.03	7.60	
	40	3.87 ± 0.03	18.35	
	80	3.72 ± 0.04	21.52	
	160	3.28 ± 0.02	30.80	
PFHxA	0	4.17 ± 0.04	0	235
	20	3.36 ± 0.02	19.42	
	40	3.00 ± 0.01	28.06	
	80	2.82 ± 0.03	32.37	
	160	2.53 ± 0.02	39.33	
PFOA	0	3.78 ± 0.03	0	122
	20	2.91 ± 0.02	23.02	
	40	2.20 ± 0.01	41.80	
	80	1.97 ± 0.04	47.88	
	160	1.75 ± 0.03	53.70	
PFDA	0	2.39 ± 0.05	0	170
	20	2.28 ± 0.03	4.60	
	40	1.88 ± 0.03	21.34	
	80	1.44 ± 0.04	39.75	
	160	1.38 ± 0.02	42.26	

*k*: the growth rate constant; *I*: the inhibitory ratio; *IC*_50_: half inhibitory concentration.

**Table 2:** Effect of combined exposure to PFCAs and copper in *k*, *I*, and *IC*_50_.

Sample	PFCAs (mg/L)	Cu^2+^ (mg/L)	*k* (×10^−2^ min^−1^)	*I* (%)	*IC*_50_ (mg/L)
PFBA	0	0	6.46 ± 0.04	0.00	228
	0	5	6.25 ± 0.05	3.25	
	20	5	5.92 ± 0.04	8.36	
	40	5	5.37 ± 0.04	16.87	
	80	5	4.98 ± 0.05	22.91	
	160	5	4.10 ± 0.03	36.53	
PFHxA	0	0	7.24 ± 0.05	0.00	156
	0	5	6.20 ± 0.02	14.36	
	20	5	5.97 ± 0.03	17.54	
	40	5	5.45 ± 0.06	24.72	
	80	5	4.65 ± 0.05	35.77	
	160	5	3.65 ± 0.05	49.59	
PFOA	0	0	6.46 ± 0.04	0.00	103
	0	5	6.49 ± 0.05	−0.46	
	20	5	4.24 ± 0.03	34.37	
	40	5	3.42 ± 0.04	47.06	
	80	5	3.11 ± 0.04	51.86	
	160	5	2.98 ± 0.02	53.87	
PFDA	0	0	7.60 ± 0.05	0.00	167
	0	5	7.37 ± 0.06	3.03	
	20	5	5.42 ± 0.04	28.68	
	40	5	4.93 ± 0.03	35.13	
	80	5	4.79 ± 0.05	36.97	
	160	5	3.86 ± 0.03	49.21	
